# C3HC4-Type RING Finger Protein *Nb*ZFP1 Is Involved in Growth and Fruit Development in *Nicotiana benthamiana*

**DOI:** 10.1371/journal.pone.0099352

**Published:** 2014-06-05

**Authors:** Wenxian Wu, Zhiwei Cheng, Mengjie Liu, Xiufen Yang, Dewen Qiu

**Affiliations:** The State Key Laboratory for Biology of Plant Disease and Insect Pests, Institute of Plant Protection, Chinese Academy of Agricultural Science, Beijing, China; George Washington University, United States of America

## Abstract

C3HC4-type RING finger proteins constitute a large family in the plant kingdom and play important roles in various physiological processes of plant life. In this study, a C3HC4-type zinc finger gene was isolated from *Nicotiana benthamiana*. Sequence analysis indicated that the gene encodes a 24-kDa protein with 191 amino acids containing one typical C3HC4-type zinc finger domain; this gene was named *NbZFP1*. Transient expression of pGDG-*NbZFP1* demonstrated that *Nb*ZFP1 was localized to the chloroplast, especially in the chloroplasts of cells surrounding leaf stomata. Virus-induced gene silencing (VIGS) analysis indicated that silencing of *NbZFP1* hampered fruit development, although the height of the plants was normal. An overexpression construct was then designed and transferred into *Nicotiana benthamiana*, and PCR and Southern blot showed that the *NbZFP1* gene was successfully integrated into the *Nicotiana benthamiana* genome. The transgenic lines showed typical compactness, with a short internode length and sturdy stems. This is the first report describing the function of a C3HC4-type RING finger protein in tobacco.

## Introduction

Zinc finger proteins are one of the most abundant types of transcription factors in eukaryotic genomes. Zinc fingers are structurally composed of multiple cysteines and/or histidines, and zinc ions play an important role in the stability of the protein itself. The RING domain of RING finger proteins, which are members of the zinc finger family, was first identified as a DNA-binding motif in the transcription factor TFIIIA from *Xenopus laevis* [1]. In addition to DNA, RING domains also bind RNA, protein, or lipid substrates. The RING motif is relatively small and consists of four pairs of ligands that bind two ions [2]. C3HC4-type RING finger proteins are involved in numerous cellular processes, including transcription, signal transduction, and recombination. Functions attributed to the RING domain itself include protein-protein interactions and ubiquitination. Most RING finger proteins are E3 ubiquitin ligases that mediate the transfer of ubiquitin to target proteins and play important roles in diverse aspects of cellular regulation in plants [3].

C3HC4-type RING finger proteins have been studied on a genomic scale in *Arabidopsis* and rice. *Arabidopsis* RING finger proteins with predicted or known biological functions include *At*COP1 (light) and *At*COP1-interacting protein 8 (AtCIP8; photomorphogenesis) [4], *At*TED3 (light signaling) [5], *At*RMA1 (secretory pathway) [6], *At*PEX10 and *At*PEX12 (peroxisome biogenesis) [7], *At*PRT1 (N-end rule pathway) [8], *At*XB3 (root development) [9], *At*HUB1 and *At*HUB2 (chromatin modifications) [10], and *At*SDIR1 (stress tolerance) [11]. C3HC4-type RING finger genes have been identified in rice, including *Os*COP1 (*Os*RHC11), *Os*COIN1 (*Os*RHC13), *Os*XB3.1 (*Os*RHC24), and *Os*RHC1. *Os*COP1 is a component of the signal transduction pathway that links light signals to plant development and is the most well-studied C3HC4-type RING finger protein. *Os*COP1 functions as an E3 ubiquitin ligase, which targets photomorphogenesis-promoting transcription factors for ubiquitylation and degradation [4]. Two other C3HC4-type RING finger genes, OsRHC24 and OsRHC1, are related to disease resistance [9].

In our previous study, we used Hrip1, which can result in the formation of necrotic lesions that mimic a typical hypersensitive response and apoptosis-related events including DNA laddering [12], as a bait protein, yeast two hybrid system was performed and five candidate interacting proteins were screened. During the process of further verification about the interaction between Hrip1 and candidate proteins, interestingly, one of the five candidate interacting proteins, which is involved in fruit development in *Nicotiana benthamiana*, in the meanwhile, after blast in NCBI and sol genomics network, there is no relevant report about this gene. In the present study, we emphasized the gene which is involved in fruit development in *Nicotiana benthamiana*, a pair of primers were designed according to the results of yeast two hybrid and sol genomics network information and C3HC4-type RING finger gene was obtained from a tobacco cDNA, this new gene was named *NbZFP1*. Prokaryotic expression of *NbZFP1* in vitro and subcellular localization of *NbZFP1* in tobacco were performed. The function of *NbZFP1* was analyzed by tobacco rattle virus (TRV) based on Virus-induced gene silencing system and over expression in tobacco. This research provides a foundation of the molecular mechanisms of C3HC4-type RING finger proteins in tobacco.

## Materials and Methods

### Plasmids and Bacterial Strains

The prokaryotic expression vector pGEX-6P-2 was purchased from Amersham Biosciences (Pittsburgh, pennsylvania state, USA). pGEX-6P-2 contains the coding region for glutathione S-transferase (GST). The plant expression vector pBI121, the subcellular localization vector pGDG, and *Agrobacterium GV3101* were derived from lab stocks. *E. coli* strain BL21 (DE3) pLysS was purchased from TransGen Biotech Inc. (China). The VIGS system vectors were a gift from Yule Liu, Tsinghua University.

### Plant Cultivation

Tobacco seeds were germinated on 1/2 MS medium in a growth chamber that was maintained at 25°C with 12 h of light and 12 h of darkness. Following germination, the seedlings were transferred to an autoclaved soil mix containing 1:3 (v/v) high-nutrient soil and vermiculite in 8×7.5×7.5-cm pots. One plant per pot was kept in the growth chamber at 25°C with 50% humidity and 16 h of light. The plants were watered on alternate days.

### *NbZFP1* Cloning and Construction of the Prokaryotic Expression Plasmid

Total RNA of *Nicotiana benthamiana* was extracted using a plant tissue RNA extraction reagent (TransGen Biotech, Beijing, China), and mRNA was used to synthesize first-strand cDNA. *NbZFP1* was amplified from first-strand cDNA of *Nicotiana benthamiana* via polymerase chain reaction (PCR) using the sense primer P1 (5′-CGGGATCCATGTCACTTTCTGGTCGT-3′) and the antisense primer P2 (5′-GCGTCGACTCAGGTTTTCAGCCCTGT-3′). The PCR thermocycling protocol was as follows: 95°C for 5 min, followed by 30 cycles of 95°C for 30 s, 60°C for 30 s, and 72°C for 1.5 min, with a final extension of 72°C for 10 min. The PCR product was visualized by ethidium bromide-containing agarose gel electrophoresis (1% agarose, 100 V for 20 min) and subsequent UV transillumination. The PCR product was then purified and digested with BamHI and SalI, inserted into the pGEX-6P-2 vector, and transformed into BL21(DE3) pLysS. Positive colonies were selected on Luria-Bertani (LB) agar plates containing ampicillin (100 µg/ml) and screened by direct colony PCR. The extracted plasmid, designated pGEX-6p-2-*NbZFP1*, was subjected to DNA sequencing by Beijing Genomics Institution, Beijing, China.

### Expression and Purification of the Recombinant Protein

The cells transformed with pGEX-6p-2-*NbZFP1* were cultured in LB medium containing ampicillin (100 µg/ml) at 37°C with shaking for eight hours. Isopropyl β-D-thiogalactoside (IPTG) was then added to a final concentration of 0.2 mM to induce expression at 16°C for 8 h. The culture transformed with the empty pGEX-6P-2 vector was used as a control. The bacteria were pelleted at 5000 g for 20 min at 4°C. The pellets were resuspended in buffer I (50 mM Tris and 200 mM NaCl, pH 8.0), and the cells were broken by sonication. After adequate sonication, the broken cells were pelleted at 12000 rpm for 1 h at 4°C, and the supernatant was collected. At this point, the samples containing pGEX-6p-2-*NbZFP1* and empty pGEX-6P-2 were ready to be purified. Because pGEX-6P-2 contains the coding region for glutathione S-transferase (GST), we used GST affinity purification technology to purify GST-*Nb*ZFP1 and GST alone in the first step. After desalination, ion exchange chromatography was applied in the next purification step [13]. Both samples were subjected to sodium dodecyl sulfate-polyacrylamide gel electrophoresis (SDS-PAGE) analysis.

### Subcellular Location of the *Nb*ZFP1 Protein

The plasmid pGEX-6P-2-*NbZFP1* was used as a template to amplify *NbZFP1* by PCR with specific primers containing BamHI and SalI restriction enzyme sites. The product was digested with BamHI and SalI and cloned into pGDG that was cut with BamHI and SalI. After successful construction of pGDG-*NbZFP1*, we transformed the plasmid into *Agrobacterium GV3101* using the freeze-thaw method [14]. *Agrobacterium GV3101* harboring pGDG-*NbZFP1* was grown in culture until the optical density of the culture reached 1.0 at 600 nm. The bacteria were pelleted at 5000 g for 15 min at room temperature. The pellets were resuspended in buffer (10 mM MES, 10 mM MgCl_2_, 200 mM acetosyringone, pH 5.6). Bacterial suspensions were then maintained at room temperature for 2–3 h. Infiltrations were performed by gently inserting a 1-ml disposable syringe into the abaxial surface of fully expanded *Nicotiana benthamiana* leaves that were approximately 2.5 cm wide at the mid-leaf and slowly depressing the plunger [15]. Following agroinfiltration, the plants were maintained in a growth chamber at 25°C with a 16/8 h light/dark photoperiod. The leaves were examined by microscopy between 40 h and 90 h post-infiltration.

### VIGS Technique for Silencing *NbZFP1* in *N. benthamiana*

The VIGS system includes the pTRV1 and pTRV2 vectors. Two adaptors with PstI restriction sites were inserted into the pTRV2 vector. This vector allows the insertion of gene silencing fragments by ligation-independent cloning (LIC) [16]. The *NbZFP1* C3HC4-type RING finger domain gene, referred to as *NbZFP1-A1*, was amplified with the primers 5′-CGACGACAAGACCCTTGCTGTGTTTGTCAGGAA-3′ and 5′-GAGGAGAAGAGCCCTTCAGGTTTTCAGCCCTGT-3′. A version of the *NbZFP1* gene lacking the C3HC4-type RING finger domain, referred to as *NbZFP1-A2*, was amplified with the primers 5′-CGACGACAAGACCCTATGTCACTTTCTGGTCGT-3′ and 5′-GAGGAGAAGAGCCCTTGGCTCAACATCCACAGG-3′. The PCR products were purified with polyethylene glycol/MgCl_2_ to remove any nonspecific PCR products and primers. A total of 50 ng of purified PCR product was treated with T4 DNA polymerase (New England Biolabs) in 1× reaction buffer containing 5 mM dATP and dithiothreitol at 22°C for 30 min followed by 20 min of inactivation of T4 DNA polymerase at 70°C. The TRV2-LIC vector was then digested with PstI and similarly treated with T4 DNA polymerase; however, dTTP replaced dATP [17]. A total of 50 ng of treated PCR product and TRV2-LIC vector were mixed and incubated at 65°C for 2 min and subsequently at 22°C for 10 min. Then, 6 µl of the mixture was transformed into *E. coli DH5α* competent cells, and the transformants were tested by colony PCR.

Sequence-validated pTRV2-*NbZFP1-A1* and pTRV2-*NbZFP1-A2* plasmids were each introduced into *Agrobacterium tumefaciens* strain *GV3101* by the freeze-thaw method [14]. Overnight cultures were grown at 28°C in the appropriate antibiotic selection medium. On the following day, the cultures were spun down, and the cells were resuspended in infiltration medium (10 mM MES, 10 mM MgCl_2_, 200 mM acetosyringone, pH 5.6), adjusted to an OD_600_ of 1, and incubated at room temperature for 3 h. *Agrobacterium* cultures containing pTRV1 and pTRV2 were mixed at a 1:1 ratio and used to infiltrate plants at the 4-leaf stage using a 1-ml needle-less syringe [18]. Total RNA was extracted from the leaves or flowers of wild-type and VIGS *N. benthamiana* plants using the RNeasy plant mini kit (Qiagen). First-strand cDNA was synthesized using 1 mg of total RNA, gene-specific primers, and SuperScript reverse transcriptase (Invitrogen) according to the manufacturer’s protocol. The expression level of *NbZFP1* was monitored by real-time PCR, and the *Actin* gene was amplified as a quantitative control. qPCR was conducted with iQ SYBR Green Supermix (Bio-Rad, Hercules, CA, USA) and an iCycler (Bio-Rad) according to the manufacturer’s instructions.

### Tobacco Transformation

Primer 5.0 was used to design the sense primer, P1 (5′-CGGGATCCATGTCACTTTCTGGTCGT-3′), and the antisense primer, P2 (5′-GCGTCGACTCAGGTTTTCAGCCCTGT-3′). The primers were then used to amplify the ORF of *NbZFP1* by polymerase chain reaction (PCR). Primers P1 and P2 contained BamHI and SalI restriction sites. The PCR products and pBI121 vector were double digested, adapter ligation was performed, and the ligation products were then transformed into *DH5α* cells. Positive colonies were selected on Luria-Bertani (LB) agar plates containing kanamycin (50 µg/ml) and screened by direct colony PCR. The fusion vector pBI121-*NbZFP1* was transformed into *Agrobacterium GV3101*. *Agrobacterium GV3101* harboring pBI121-*NbZFP1* was used to transform *N. benthamiana* according to the method proposed by Horsch et al [19], and the transformed plants were then transferred to soil for seed setting. T_1_ progeny plants from the seeds of independent T_0_ transformants were grown on 1/2 MS medium with kanamycin selection, and green plantlets with roots were transferred to soil.

PCR analysis of T_0_ plants was performed for the detection of putative transgenic tobacco plants. Genomic DNA from fresh, fully expanded tobacco leaves was used for the PCR analysis. pBI121 harbors a *gus* gene; thus, amplification of a specific fragment of the *gus* gene was performed by adjusting the PCR conditions for 30 cycles. The primers forward-F (5′-ATGGTCCGTCCTGTAGAAACC-3′) and primers reverse-R (5′-GACTGCCTCTTCGCTGTACAG-3′) were used for amplification of the specific *gus* fragment. After positive identification of transgenic plants, we performed Southern blotting to determine the *NbZFP1* copy numbers according to the protocol provided with the DIG High Prime DNA Labeling and Detection Starter Kit II (Amersham Biosciences).

30 Single copy T_1_ progeny plants were grown on 1/2 MS medium containing kanamycin, and 30 wild progeny plants were grown without antibiotic as a control. Green plantlets with roots were transferred to pots containing soil. The height of the plants were measured beginning at the four-leaf stage in 10-day intervals. The internode lengths of wild-type and transgenic tobacco were measured at the sixty day after four-leaf stage.

### Statistical Analysis

The data were analyzed separately for each experiment with SPSS 16.0 software. The means were compared using Tukey’s HSD test. The significance of the different between the means of wild-type and over-expression tobacco in the same growth condition were calculated by chi square test using SPSS 16.0 software. ^***^ indicate significance at the 0.001 of confidence level.

## Results

### *NbZFP1* Cloning

Using a pair of specific primers, the gene we have designated as *NbZFP1* was cloned from *N. benthamiana* by RT-PCR. The length of the full open reading frame (ORF: GenBank accession number KJ169550) for this gene is 576bp.

### Phylogenic Analysis

**Figure 1:**
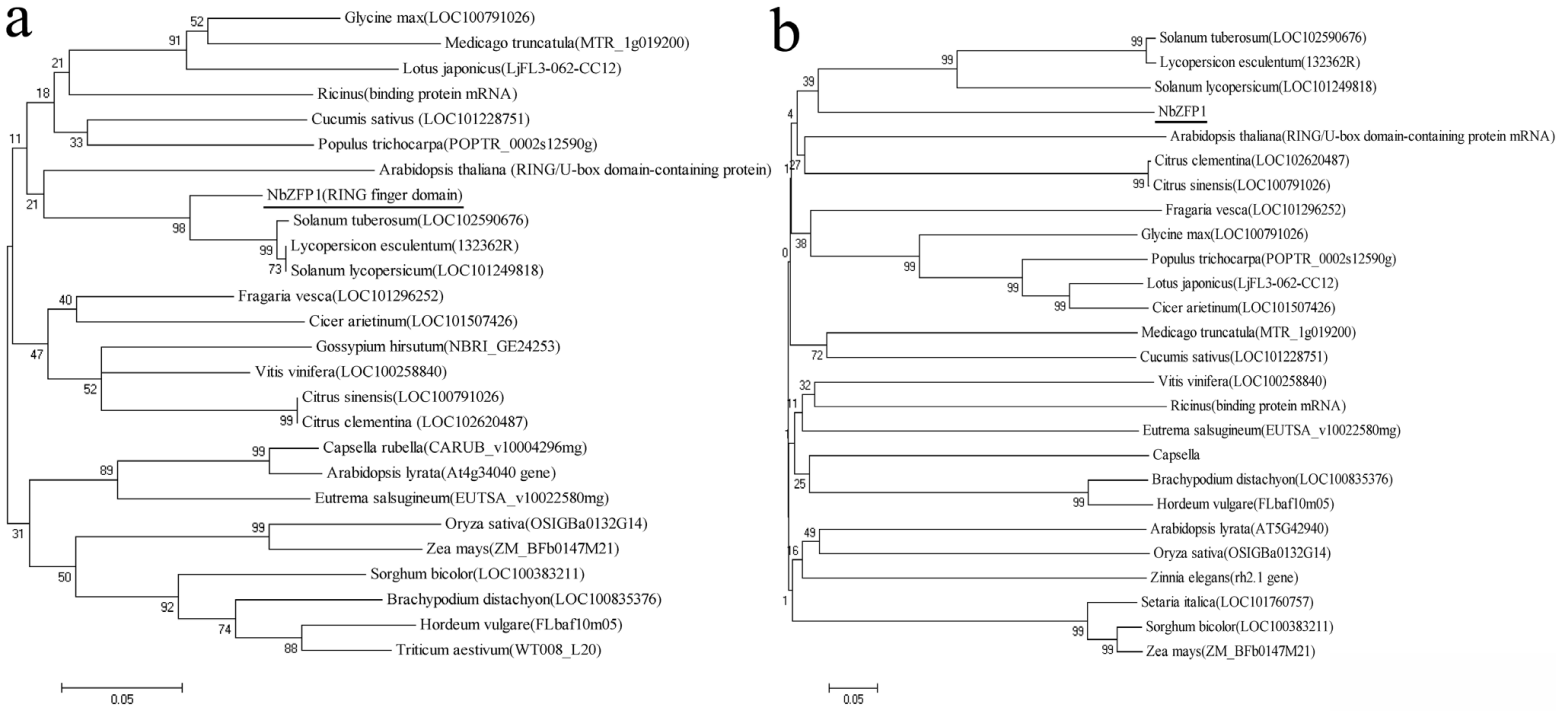
Phylogenetic analysis of the relationships between *NbZFP1* and C3HC4-type RING finger genes in other species. We selected twenty-five typical C3HC4-type RING finger genes with high similarity from different species, and we analyzed the similarity of their RING finger domains and full-length sequences with *NbZFP1*. Phylogenetic trees were generated by the maximum likelihood (ML) method using MEGA4. Bootstrap values from 1000 replicates are indicated at each branch. (a) A maximum likelihood (ML) tree of *NbZFP1* and genes from other species was constructed based on RING finger domain sequences. (b) A maximum likelihood (ML) tree of *NbZFP1* and genes from other species was constructed based on full-length nucleotide sequences.

To define the relationships between *NbZFP1* and C3HC4-type RING finger genes in other species, phylogenetic analyses were performed with RING finger domain sequences and full-length sequences [20]. The maximum likelihood (ML) tree [21] of the C3HC4-type RING finger domain-containing motif suggested that C3HC4-type RING finger genes are highly conserved in 26 species. In addition, *NbZFP1* is more closely related to the C3HC4-type RING finger genes of *Solanum tuberosum*, *Lycopersicon esculentum,* and *Solanum lycopersicum* than those of other species (Figure 1a). The tree of full-length sequences of C3HC4-type RING finger genes showed that the similarities between *NbZFP1* and the C3HC4-type RING finger genes in the other 25 species are not high (Figure 1b).

### Purification and Detection of Recombinant *Nb*ZFP1 Protein

**Figure 2:**
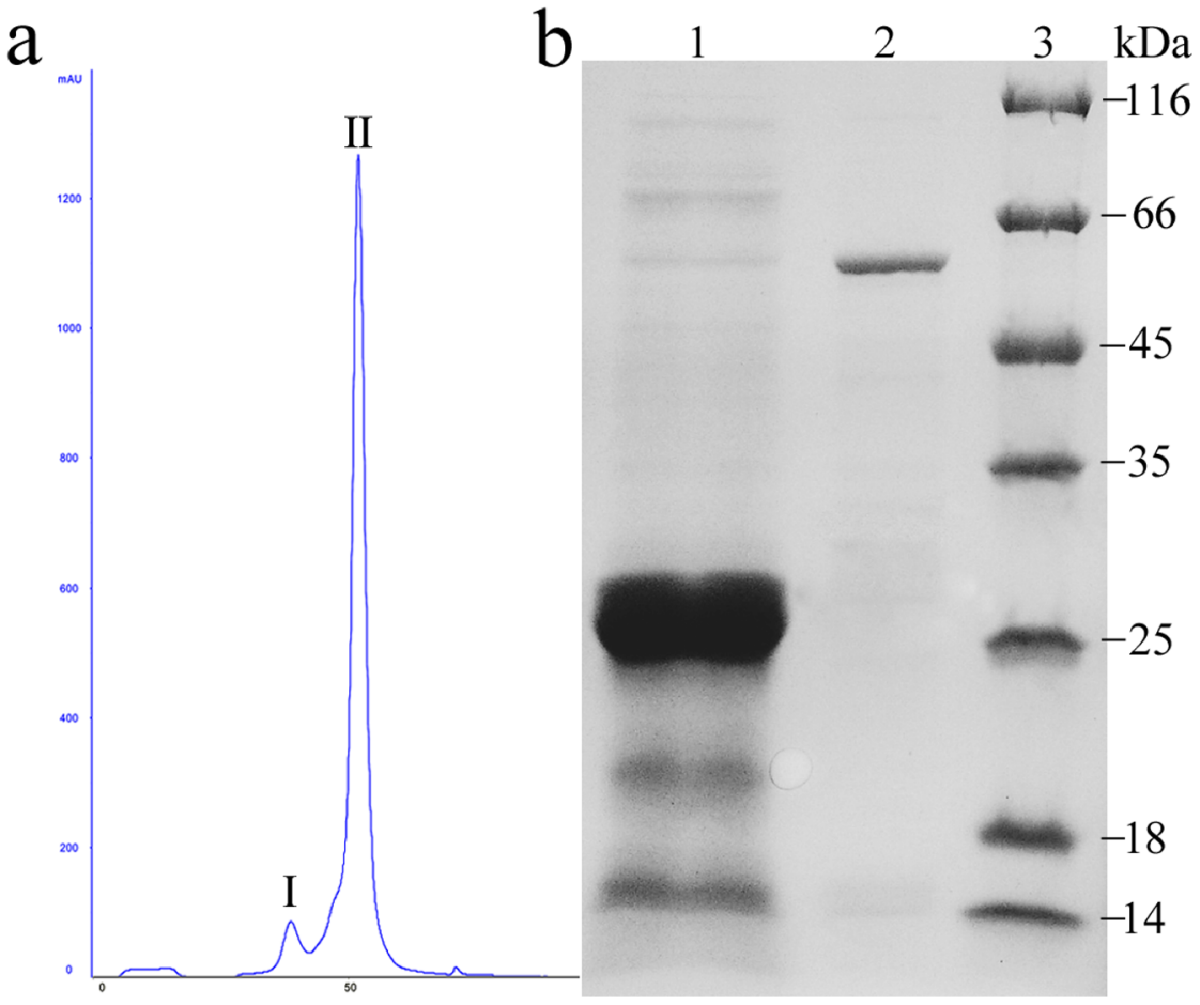
Purification and detection of recombinant protein. The *Nb*ZFP1 protein was expressed in *E. coli* and purified with affinity chromatography (GSTrap™ HP column) and ion exchange chromatography (HiTrap Q HP column). The purified protein showed a single band on SDS-PAGE stained with Coomassie Brilliant Blue R-250. (a) Ion exchange chromatography of a concentrated solution loaded onto a HiTrap Q HP column. (b) SDS-PAGE analysis of the recombinant protein. Lane 1, GST protein after affinity chromatography purification. Lane 2, protein peak II as designated in (a). Lane 3, protein marker.

*NbZFP1* was cloned into the prokaryotic expression vector pGEX-6p-2, and the resulting recombinant plasmid, pGEX-6p-2-*NbZFP1*, was confirmed by colony PCR and DNA sequencing. The recombinant plasmid was then transformed into *E. coli BL21* (DE3) to express the recombinant protein. The expressed protein was soluble in *E. coli* and was purified with a GSTrap HP column followed by a HiTrap desalting column and a HiTrap Q HP column (Figure 2a). The molecular weight of the GST tag is 26 kDa, and SDS-PAGE analysis showed a single band corresponding to the purified recombinant protein with an approximate molecular mass of 50 kDa (Figure 2b). This result indicated that the *Nb*ZFP1 protein was expressed in *E. coli*.

### Subcellular Localization of the *Nb*ZFP1 Protein

**Figure 3:**
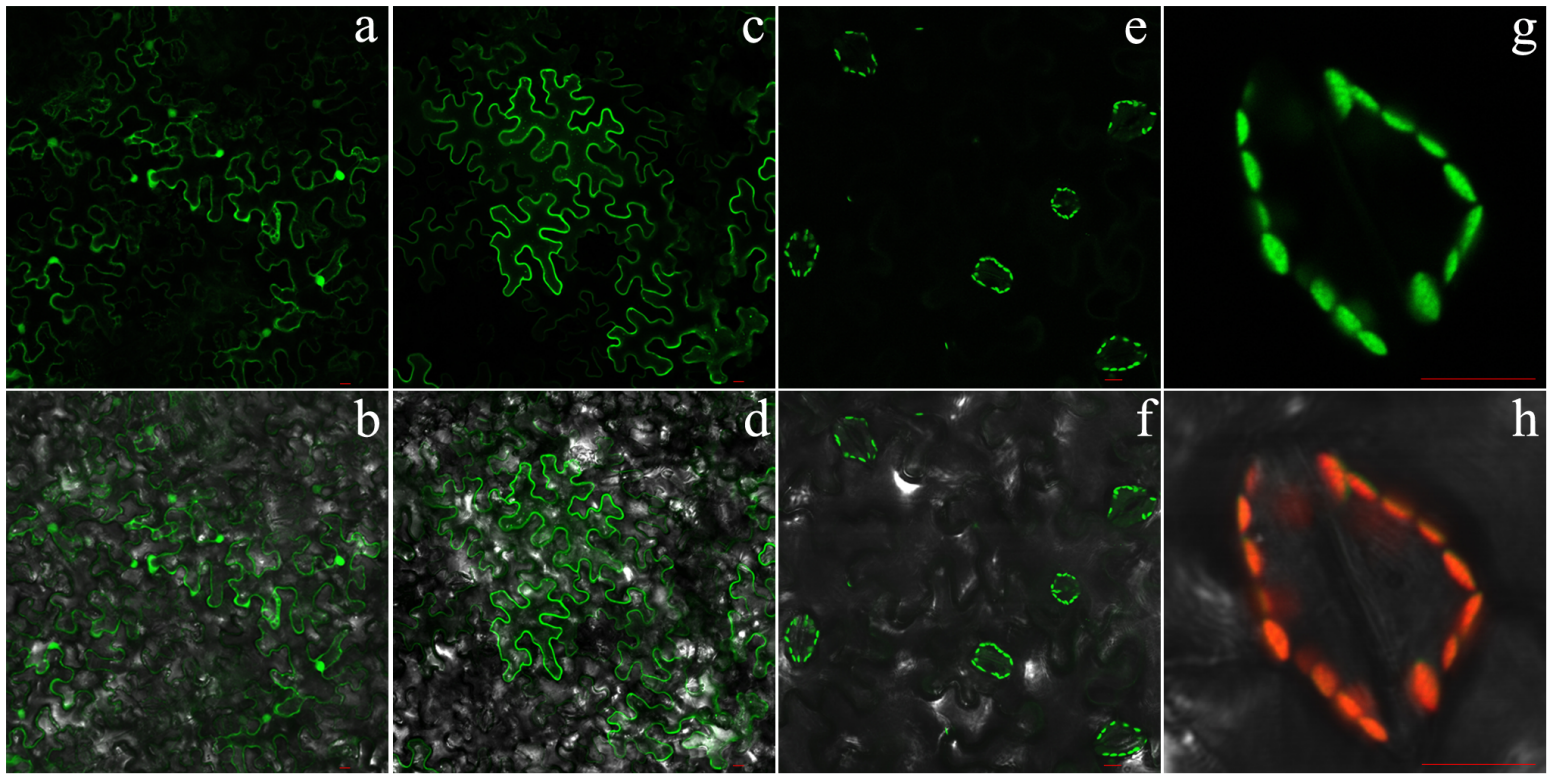
The results about subcellular localization of the *Nb*ZFP1 protein. Laser-scanning confocal micrographs showing the fluorescence of leaf cells following infiltration with *Agrobacteria* carrying pGDG, pGDG-*Rac1*, or pGDG-*NbZFP1* plasmids expressing GFP, Rac1-GFP, and *Nb*ZFP1-GFP proteins, respectively. Scale bar = 10 µm. (a) and (b) GFP expressed from pGDG; (c) and (d) GFP expressed from pGDG-Rac1; (e), (f), (g), and (h) Fluorescence expressed from pGDG-*NbZFP1*. (a), (c), (e), and (g) show the green channel; (b), (d), and (f) show an overlay of the bright-field and green channels; (h) shows an overlay of the bright-field, green, and red channels.

To confirm the subcellular localization of the *Nb*ZFP1 protein, we constructed a recombinant vector, pGDG-*NbZFP1*, for transient expression. The empty pGDG vector was used as a negative control. Because the Rac1 protein was shown to be localized to the plasma membrane [22], the pGDG-*Rac1* vector was constructed for expressing Rac1-GFP as a positive control. *Agrobacterium GV3101* containing pGDG, pGDG-*Rac1*, or pGDG-*NbZFP1* was used to infiltrate healthy tobacco plants. Laser scanning confocal micrographs showed that GFP alone localized throughout the whole cell, including the plasma membrane, nucleus, and cytoplasm (Figure 3a and Figure 3b). The fusion protein Rac1-GFP was localized to cell membranes only (Figure 3c and Figure 3d). The protein of interest, *Nb*ZFP1-GFP, was located in the chloroplast, especially in the chloroplasts of cells surrounding leaf stomata (Figure 3e–h).

### Developmental Phenotypes Revealed by Silencing *NbZFP1*

TRV-mediated VIGS is an effective tool for assessing the functions of genes in the reproductive tissues of plants [23]. Silencing of phytoene desaturase (PDS) leads to the inhibition of carotenoid synthesis, causing the plants to exhibit a photobleached phenotype [24]. Thus, we assessed the gene silencing efficiency of our TRV-VIGS clones by suppressing the expression of the PDS gene in *N. benthamiana*. The PDS suppression phenotype was visible at 10 days post-infiltration in the upper leaves of the plant and persisted indefinitely. This result indicated that the TRV-VIGS system could be successfully used to induce silencing of other desirable endogenous plant genes [18].

**Figure 4:**
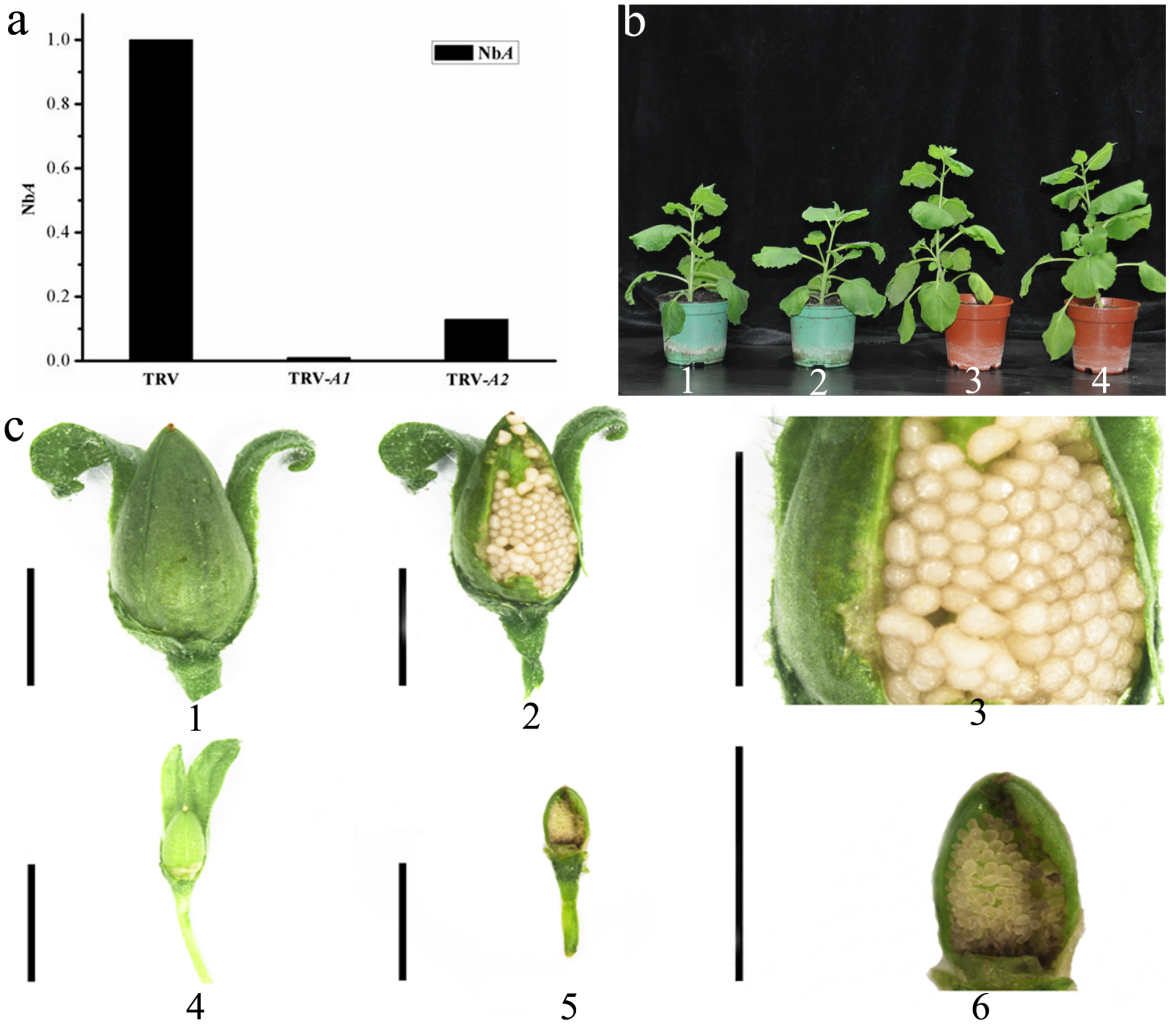
Characterization of VIGS strains by silencing of *NbZFP1* compared with controls. (a) Effect of VIGS on *NbZFP1* (in this graph, *NbA*) transcription in *N. benthamiana*. TRV: The expression level of *NbZFP1* in plants infected with TRV alone; TRV-*A1*: The expression level of *NbZFP1* in plants infected with TRV-*A1* (*A1* is the C3HC4-type RING finger domain gene *NbZFP1*); TRV-*A2*: The expression level of *NbZFP1* in plants infected with TRV-*A2* (A2 is *NbZFP1* lacking the C3HC4-type RING finger domain). (b) The phenotype of VIGS-silenced *NbZFP1* and wild type strains. b1, *NbZFP1-*silenced plant at 20 days after the four-leaf stage; b2, wild type plant at 20 days after the four-leaf stage; b3, *NbZFP1-*silenced plant at 40 days after the four-leaf stage; b4, wild type plant at 40 days after the four-leaf stage. (c) The fruit phenotype of *NbZFP1-*silenced plants compared with controls. c1, c2, and c3: The fruit phenotype of non-silenced control plants. c4, c5, and c6: The fruit phenotype of *NbZFP1-*silenced plants.

We then cloned two different fragments of *NbZFP1* gene separately into the TRV2-LIC vector for silencing. The first of these fragments was obtained from the *NbZFP1* C3HC4-type RING finger domain gene *NbZFP1-A1*, while the other fragment was obtained from *NbZFP1-A2*, which is a version of *NbZFP1* that lacks the C3HC4-type RING finger domain. We infiltrated plants at the four-leaf stage with a 1:1 mixture of TRV1 and the TRV2-LIC-*NbZFP1* fragment for each of the clones, and we monitored the infiltrated plants throughout their entire lifespan. We then determined the degree of silencing of *NbZFP1* fragments by qPCR. The results revealed a greater than 90% reduction in transcript levels in the silenced plants (Figure 4a). Silencing *NbZFP1* resulted in fruits that were smaller than those of the TRV-infected control (Figure 4c), although no other phenotypic defects were observed (Figure 4b).

### Generation of *NbZFP1* Transgenic Tobacco Plants

**Figure 5:**
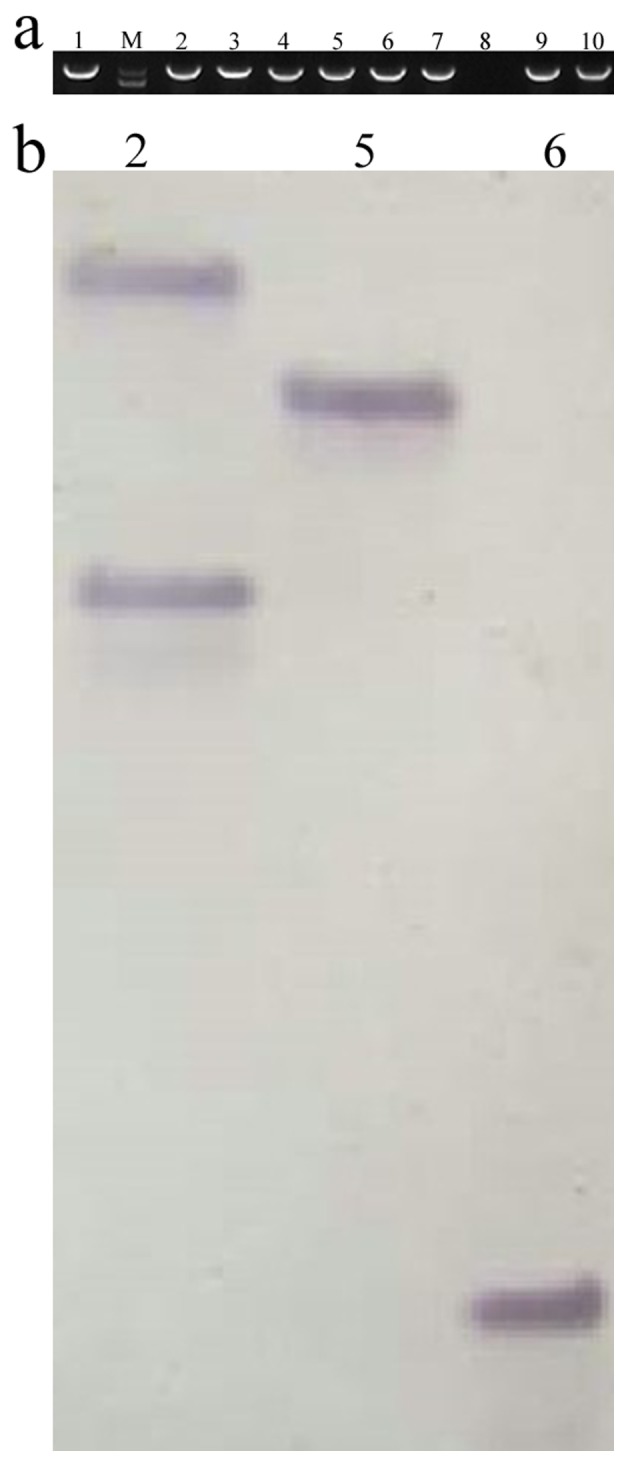
Identification of transgenic tobacco plants. Fresh leaves (100 mg) from T_1_ plants were collected, and genomic DNA was extracted. A specific fragment of the *gus* gene (approximately 1000 bp in length) was amplified to identify the transgenic lines. (a) PCR identification of transgenic tobacco plants. M, DL8000 marker; 1, positive control; 2–10, transgenic tobacco lines. (b) Southern blot analysis of transgenic tobacco plants. 2, Transgenic line 2; 5, Transgenic line 5; 6, Transgenic line 6.

The plant expression vector pBI121 containing *NbZFP1* was transformed into tobacco via the *Agrobacterium*-mediated method [19]. Nine independent kanamycin-resistant *NbZFP1* transgenic tobacco lines were obtained. These transgenic tobacco plants were verified by PCR, and eight transgenic lines were positive (Figure 5a). We randomly selected 3 transgenic plants and performed a Southern blotting experiment, which showed that *NbZFP1* was successfully integrated into all 3 transgenic tobacco plants and two of the lines had a single copy of *NbZFP1* (Figure 5b).

### Phenotypic Effect of *NbZFP1* in Transgenic Lines

**Figure 6:**
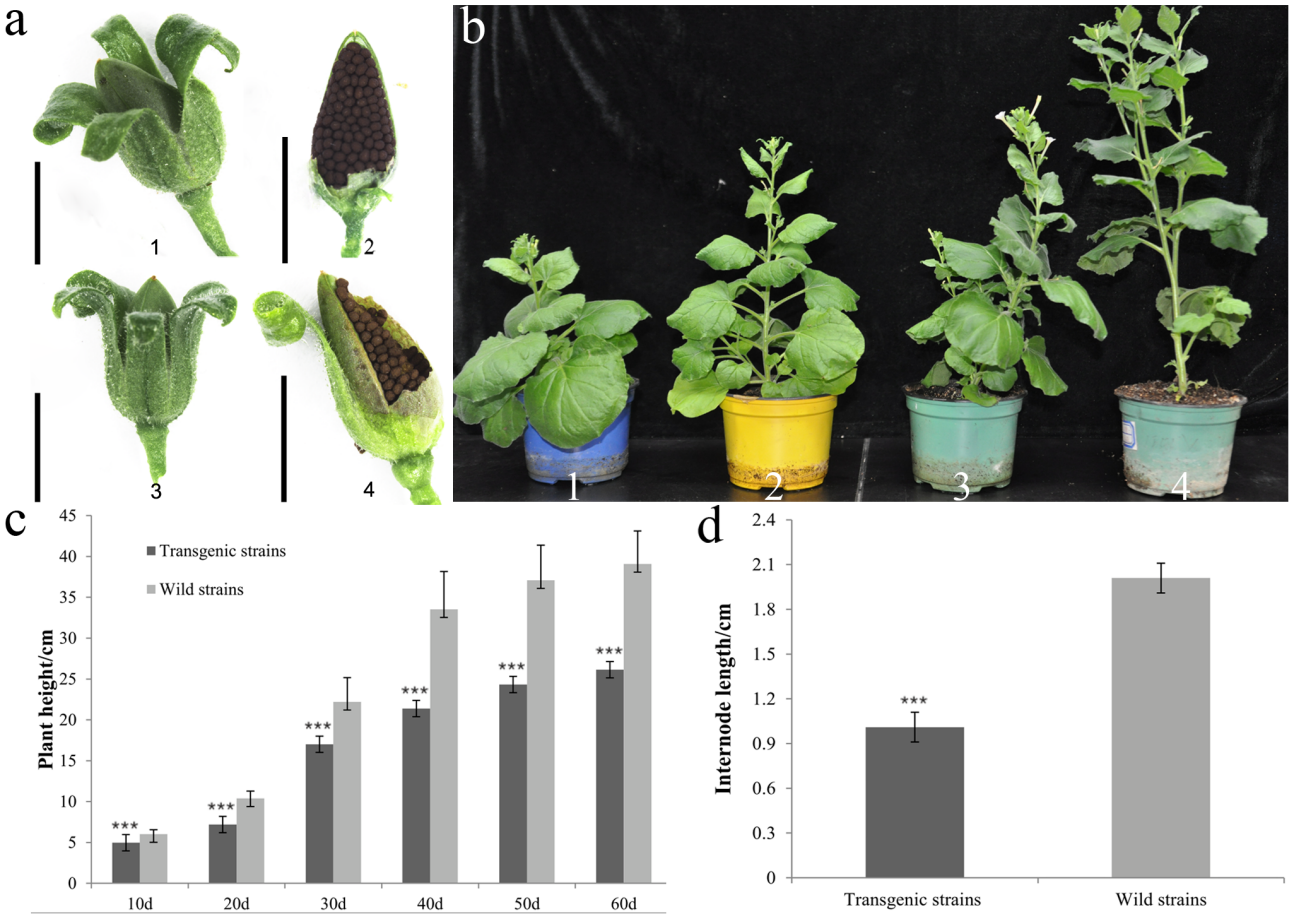
Characterization of T1 single copy lines compared with controls. (a) The fruit phenotype of transgenic lines compared with wild type plants. a1 and a2, the fruit phenotype of transgenic plants. a3 and a4, the fruit phenotype of wild type plants. (b) The phenotype of T_1_ single copy lines and wild type lines. b1, T_1_ generation single copy transgenic strains at 20 days after the four-leaf stage; b2, wild type strains at 20 days after the four-leaf stage; b3: T_1_ single copy transgenic strains at 50 days after the four-leaf stage; b4, wild type strains at 50 days after the four-leaf stage. (c) The height of single copy *NbZFP1* transgenic plants compared with the height of wild type plants at different times. (d) The internode lengths of single copy *NbZFP1* transgenic plants compared with the wild type internode length, 30 plants were measured. Asterisks indicate significant differences from the wild type: ^***^P<0.001.

We selected T_0_ plants with a single copy of *NbZFP1* and obtained their seeds. These seeds were then sown on 1/2 MS medium with kanamycin selection. Wild type plants were grown on 1/2 MS medium without antibiotic. 30 wild-type and transgenic plantlets with roots were transferred to soil, respectively. The height of the plants were measured 10 days after the plants had reached the four-leaf stage, the internode lengths of wild-type and transgenic tobacco were measured at the sixty day after four-leaf stage. We found that transgenic lines were shorter than wild type lines after the four-leaf stage, and the regenerated plants were compact with short internodes and sturdy stems (Figure 6b, Figure 6c, Figure 6d). However, the phenotype of transgenic fruits was normal (Figure 6a).

## Discussion

C3HC4-type RING finger proteins play different roles in diverse physiological processes. Many C3HC4-type RING finger genes in plants have been shown to play important roles in the regulation of growth and development [25]. The C3HC4-type RING finger proteins can be divided into three types according to their role. Because the specificity of the ubiquitin proteasome pathway is determined by E3 ligases, the various functions of C3HC4-type RING finger proteins may be explained by the fact that they act as E3 ligases with different targets in diverse physiological processes. Indeed, C3HC4-type RING finger proteins can regulate many cellular processes, including homeostasis, development, cell division, growth, hormone responses, and stress responses [26]. Some C3HC4-type RING finger genes function as cis-elements and play key roles in the transcriptional regulation of genes controlling various biological processes, including abiotic stress responses [27]. In addition, C3HC4-type RING finger genes also participate hormonal regulation. Phytohormones are the most important signaling molecules in plants, and the concentration of a given phytohormone influences plant growth and development. For example, auxin regulates cell processes and promotes cell elongation. GAs are a large family of plant hormones, some of which are bioactive growth regulators that control seed germination, stem elongation, and flowering [28].

In this study, we provide a look at the C3HC4-type zinc finger protein of tobacco. Sequence analysis indicated that the gene encodes a 24-kDa protein with 203 amino acids, and we refer to this gene as *NbZFP1*. The results of VIGS analysis show that the C3HC4-type zinc finger protein had a clear effect on the process of tobacco seed pod development, although the height of the plants subjected to VIGS treatment was normal. When *NbZFP1* was transformed into tobacco, we found that the fruit phenotype of T_1_ transgenic lines containing a single copy of *NbZFP1* was normal, although these lines were characterized by compactness, short internodes, and sturdy stems. We next examined the subcellular localization of *Nb*ZFP1, and we found that *Nb*ZFP1 was localized in the chloroplast, especially in the chloroplasts of cells surrounding leaf stoma. We therefore hypothesized that *Nb*ZFP1 has a role in stomatal movements. It is known that stomatal opening and closing is mediated by ABA-triggered changes in ion fluxes in guard cells [29], [30], [31]. ABA is considered to be an important hormone, as it plays a critical role in the response of plants to various stresses. ABA is not only a stress signal but is also required to fine-tune growth and development under non-stress conditions. The physiological processes controlled under these conditions include the regulation of growth, stomatal aperture, hydraulic conductivity, and seed dormancy [32], [33], [34]. ABA negatively influences the size of guard cells and the internode length, and it also acts together with other phytohormones, such as brassinosteroids, gibberellic acid, and auxin, in the regulation of plant growth and development [35], [36], [37]. The characteristics of the transgenic lines are consistent with the function of zinc finger proteins in other plants. For example, the EPF zinc finger protein family of *Petunia hybrida* and the SUPERMAN or NNT zinc finger protein of *Arabidopsis thaliana* are both involved in regulating the process of reproductive development [38], [39], [40] The rice zinc finger protein PROG1 is directly involved in plant growth regulation and domestication [41]. *NbZFP1* might be regulate the concentration of the phytohormone abscisic acid (ABA) through a specific mechanism, as ABA negatively regulates the internode length, or might act together with other phytohormones related to auxin signal transduction or the auxin/cytokinin signal transduction process, consequently affecting the growth and development of tobacco. However, it is currently unclear how *Nb*ZFP1 influences fruit development. Ma et al. [25] found that among 29 C3HC4-type RING finger genes in rice, 5 genes were preferentially expressed in reproductive tissues or organs. Commonly, a high level of expression or preferential expression in tissues or organs suggests that the highly/preferentially expressed gene may play an important role there. Thus, these 5 rice genes may play an important role at the reproductive stage.

Overall, C3HC4-type RING finger genes have been shown to play important roles in the regulation of growth and development in plants; however, these genes undoubtedly have many functions that have not yet been discovered. The current study provides preliminary data that requires further validation and establishes a basis for further studies. Much work remains to be carried out in the future.
